# Economic Benefits of Sponsored Clinical Trials in Cancer for the Colombian Healthcare System: A Real‐World Evidence Approach

**DOI:** 10.1002/cam4.71099

**Published:** 2025-07-31

**Authors:** Leonardo Rojas, Natalia Sánchez, Jorge Ceballos, Antonio Robles, Carlos A. Badillo, Virginia Abello, Carlos Bonilla, William A. Mantilla, Jairo Zuluaga, Gilberto Lopes, Oscar Arrieta, Andrés F. Cardona

**Affiliations:** ^1^ Luis Carlos Sarmiento Angulo Cancer Treatment and Research Center (CTIC) Bogotá Colombia; ^2^ GIGA/TERA Research Group, Luis Carlos Sarmiento Angulo Cancer Treatment and Research Center (CTIC)/El Bosque University Bogotá Colombia; ^3^ Roche Colombia Bogotá Colombia; ^4^ Division of Medical Oncology Associate Director for the Cancer Center and Medical Director for International Affairs, Sylvester Comprehensive Cancer Center Miami Florida USA; ^5^ Thoracic Oncology Unit, Instituto Nacional de Cancerologia (INCan) Mexico City Mexico

**Keywords:** cancer treatment, clinical trials, economic impact, healthcare efficiency

## Abstract

**Purpose:**

Clinical trials (CTs) are essential for the research and development of new cancer treatment technologies. Evaluating their economic impact and the potential cost savings for healthcare systems in low‐ and middle‐income countries is crucial for informing healthcare policy and decision‐making. This study estimates the economic benefits to the Colombian healthcare system from the inclusion of hematology and oncology patients in sponsored CTs.

**Methods:**

This study utilized real‐world data from the Luis Carlos Sarmiento Angulo Cancer Treatment and Research Centre (CTIC), a comprehensive cancer center in Bogotá, Colombia. Tumor types were selected based on their prevalence and economic burden. A Budget Impact Analysis was conducted following the methodology of the local Health Technology Assessment Agency, using data from five prioritized tumor types. Clinical data and associated costs were extracted from the institutional data lake, and cost‐generating events for each disease were validated by CTIC clinical experts. The estimated eligible population for phase 3 CTs was derived from literature reviews and expert opinions from CTIC clinicians. Prevalent and incident population data were obtained from the Colombian High‐Cost Account.

**Results:**

A total of 7703 potential patients were eligible for inclusion in the CTs, with an associated healthcare cost of USD 244,151,552 by 2023 (1 USD = 4325 COP). If at least 20% of these patients participated in CTs by 2023, the projected annual cost savings would be USD 48,830,310. Among the evaluated cancers, advanced prostate cancer incurred the highest costs due to its high prevalence and potential for inclusion in CTs.

**Conclusion:**

Over 5 years, potential cost savings could range from USD 244 million (assuming a 20% enrolment rate) to 1.22 billion (with 100% enrolment), alleviating financial pressures on the Colombian healthcare system. These savings would contribute to the system's long‐term financial sustainability while ensuring timely access to innovative cancer treatments.

## Graphical abstract

Sponsored clinical trials in Colombia could generate significant cost savings for the healthcare system by covering treatment expenses for cancer patients. An analysis at an oncology center estimated that with 20% patient participation, annual savings would reach USD 48.8 million, and up to USD 1.22 billion over 5 years with 100% inclusion. This would contribute to the system's financial sustainability and improve access to innovative therapies.

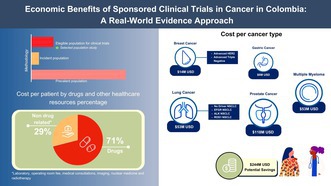

## 1 Introduction

Clinical trials (CTs) play a pivotal role in the research and development of new medical technologies, providing valuable insights into the efficacy, safety, and effectiveness of various molecules before and after regulatory approval for clinical use [1]. Globally, public health expenditures have risen due to demographic and epidemiological shifts, the adoption of new therapies, the costs associated with managing advanced‐stage diseases, improvements in healthcare quality, and population aging. In response to these challenges, health systems worldwide are exploring innovative strategies to enhance efficiency and reduce unnecessary costs while simultaneously maintaining or improving the standard of patient care [1, 2].

One approach to containing this expenditure is to enroll patients in privately sponsored CTs. In these cases, the sponsor provides the necessary resources, ensuring that the healthcare system does not bear any costs for treating study participants. The most substantial cost savings arise from the free provision of experimental drugs, patient follow‐ups, and essential interventions, including diagnostic procedures, laboratory tests, and radiologic imaging. Additionally, these trials stimulate investment in research service providers, such as clinical research organizations (CROs) [1, 3].

A hypothetical model with a higher CT participation rate could be developed to assess the impact of CT involvement on clinical outcomes, such as mortality and survival. Evidence of such a system already exists; historically, children under 15 years have had higher enrollment rates in CTs than adults [4]. Concurrently, cancer‐related mortality in children declined at an annual rate of 2.6% between 1975 and 1995 [5]. This trend suggests a direct and proportional relationship between patient inclusion in CTs and advancement in clinical and technological developments, leading to significant improvements in outcomes.

Chow et al. analyzed data from more than 550,000 patients in the California Population Cancer Registry and reported a 26% reduction in the risk of death among those who participated in CTs [6]. Similarly, Unger et al. examined 21 Southwestern Oncology Group (SWOG) CTs, which included nearly 5100 patients. They found a hazard ratio of 0.74 (95% CI = 0.64–0.84) for 2‐year overall survival compared with patients in the Surveillance, Epidemiology, and End Results (SEER) cancer registry who had a poor prognosis [7].

Despite these benefits, fewer than 5% of adult cancer patients are estimated to enroll in CTs [8], with Hispanics and African American populations being significantly underrepresented [9]. However, approximately 70% of individuals in high‐income countries report a willingness to participate in CTs, particularly in the later stages of the studies [10].

Although cost analyses related to CTs have been documented in the literature, evidence of the economic benefits of reducing healthcare resource utilization through participation in sponsored CTs remains scarce in low‐ and middle‐income countries. In Colombia, total healthcare expenditure in 2021 amounted to USD 23.4 billion [11], with approximately 21% allocated to pharmaceutical costs [12]. In 2007, the Ministry of Health and Finance established the High Cost Account (HCA), an institution responsible for managing and providing data to support risk management. The HCA facilitates coordination among various healthcare system stakeholders to improve the care of patients with high‐cost diseases, including cancer.

According to the HCA, in 2021, there were 46,870 newly reported cancer cases and 462,857 prevalent cases in Colombia [13]. Approximately 94% of both new and prevalent cases were classified as invasive. Among women, breast cancer accounted for 29.20% of all new cases and 32.37% of prevalent cases, while in men, prostate cancer comprised 21.62% of new cases and 30.28% of prevalent cases. Breast cancer had the highest standardized mortality rate at 16.1 per 100,000 inhabitants, followed by prostate cancer at 12.6 and gastric cancer at 5.0 [13]. Given this epidemiological landscape, Colombia could benefit significantly from increasing its participation in CTs, as the associated opportunity costs, such as early access to innovative therapies, investment in healthcare infrastructure, and related economic advantages, could be substantial.

Therefore, generating local data is essential to demonstrate the economic impact of CTs in our setting and to support health decision‐making. Accordingly, this study aimed to quantify the economic benefits of enrolling patients with advanced cancer (breast cancer, non‐small‐cell lung cancer, colorectal carcinoma, gastric cancer, prostate cancer, and refractory multiple myeloma) in privately sponsored phase 3 CTs within the Colombian healthcare system.

## 2 Methods

### 2.1 Budget Impact Analysis

To estimate the economic benefits of participating in sponsored CTs, we followed a previously reported Budget Impact Analysis (BIA) approach [1]. According to this methodology, two scenarios are compared: one in which CT participation reaches its full potential and another where there is no involvement in CTs [1]. In the first scenario, the sponsor assumes all direct medical costs; whereas in the second scenario, these costs are borne by a third party, which in Colombia is the General Health and Social Security System, covering 99.12% of the population [14]. We adhered to the BIA methodology established by the Colombian Health Technology Assessment Institute (IETS) [15].

### 2.2 Data Sources

We utilized real‐world data (RWD) from patients treated at the Luis Carlos Sarmiento Angulo Cancer Treatment and Research Centre (CTIC), a comprehensive treatment and research cancer centre in Bogotá, Colombia. Data on healthcare resource utilization for the selected tumors were collected from September 1, 2022, to August 31, 2023. The primary data source for each disease was the electronic health record (EHR) system used by CTIC in the EVA study, a CTIC initiative aimed at developing an integrated information platform for capturing and analyzing RWD.

### 2.3 Eligible Patients

The potential number of patients eligible to participate in CTs for each selected tumor was projected using the local HTA methodology [15]. We followed the approach outlined by Mauskopf et al. [16]. Prevalence, incidence, and CT participation probability were obtained from local databases, GLOBOCAN, published literature, and the CTIC's clinical expert opinions. The eligibility criteria are detailed in Table 1. Tumor selection was based on prevalence and economic burden. First, we identified the prevalent (> 18 years) and incident populations for each tumor in Colombia in 2023. When necessary, only females (for breast cancer) or males (for prostate cancer) were included. Next, we applied tumor‐specific eligibility criteria to refine the total target population. Finally, we estimated the eligible participants by multiplying the target population by the proportion of patients expected to enroll in CTs for each tumor.

**TABLE 1:** Patient eligibility criteria considered in the study.

Patients' eligibility criteria	
Breast cancer	HER2‐positive breast cancer independent of hormone receptor status and with metastatic disease (first‐ and second‐line therapy) Metastatic triple‐negative breast cancer (first‐ and second‐line treatment)
Gastric cancer	Metastatic gastric cancer (first‐line)
Prostate cancer	Advanced prostate cancer with or without homologous recombination repair (HRR) alterations
Non‐small cell lung cancer	Non‐oncogenic addicted non‐small cell lung cancer eligible to receive first‐line treatment with immunotherapy alone or in combination Oncogenic target non‐small cell lung cancer (EGFR, ALK, ROS1) eligible for first‐ and second‐line targeted therapy
Multiple myeloma	Multiple myeloma (first‐line including hematopoietic stem cell transplantation and refractory disease)

*Note:* Eligible patients were adults (> 18 years). Only female patients were considered to have breast cancer. Only male patients with prostate cancer were included in the study.

Abbreviations: ALK, anaplastic lymphoma kinase; EGFR, epidermal growth factor receptor; HER2, human epidermal growth factor receptor 2.

### 2.4 Statistical Analyses

The clinical experts at CTIC identified treated patients who met the eligibility criteria and validated the cost‐generating events associated with each disease. If no patients with a specific disease were treated at CTIC during the study period, we applied the IETS BIA methodology to estimate the average healthcare resource utilization per patient based on CTIC's clinical protocols. This estimated resource utilization was then integrated into CTIC's cost data to calculate the average cost per patient. When cost data were unavailable at CTIC, public databases were used as an alternative source.

The Business Intelligence Department at CTIC compiles information on drug prices, medical procedures, devices, medical supplies, and other resources needed for treatment. Given that the CTIC provides healthcare services to multiple health maintenance organizations (HMOs), cost estimates were averaged across all HMOs. The average cost per patient for each tumor was calculated by summing all cohort management‐related expenses and dividing it by the total number of identified patients. Total costs are reported as the proportion of expenditures on drugs and other healthcare resources. All results are presented in 2023 USD (1 USD = 4325 COP).

Additionally, we estimated potential savings under various enrollment scenarios, considering CT inclusion rates of 20%, 40%, 60%, 80%, and 100%.

### 2.5 Sensitivity Analysis

A deterministic sensitivity analysis was performed to assess the impact of variations in two key parameters: the prevalence of each cancer type and the cost of treatment medications. Based on this prevalence, we examined how changes in the number of eligible participants influenced outcomes, using both minimum and maximum estimates. Additionally, discount rates of 10% and 30% were applied to the medication costs.

## 3 Results

### 3.1 Eligible Patients

Population estimates indicated that 7703 patients in Colombia had the potential to undergo CTs in 2023. Table 2 presents the results categorized by each tumor type. A population‐weighted average of 21% of patients was potentially eligible for CTs, representing 0.02% of the adult Colombian population. Due to its high prevalence and incidence, advanced prostate cancer presented the greatest opportunity for patient inclusion in CTs, with 3639 eligible patients, nearly half of the total estimated potential.

**TABLE 2:** Prevalent, incident, and eligible population estimates.

Tumor	Prevalence rate	Incidence rate	Subtype	Prevalent cases by subtype	Incident cases by subtype	Eligible population for CT
Breast cancer	339.91 [13]	58.9 [17]	Advanced HER2+ BC	3778 (11.8%) [13]	182 (4%) [17]	156 (2%) [18]
			Advanced triple negative BC	4015 (12.5%) [13]	193 (4.3%) [17]	124 (1.6%) [18]
Lung cancer	9.64 [13]	2.43 [13]	No driver NSCLC	1903 (5.9%)^a^ [13, 19, 20]	687 (15.2%)^a^ [13, 19, 20]	1035 (13.4%)^a^
			EFGR NSCLC	1046 (3.3%) [13, 19, 20]	264 (5.9%)^a^ [13, 19, 20]	344 (4.5%)^a^
			ALK NSCLC	209 (0.7%)^a^ [13, 19, 20]	53 (1.2%)^a^ [13, 19, 20]	100 (1.3%)^a^
			ROS1 NSCLC	84 (0.3%)^a^ [13, 19, 20]	21 (0.5%)^a^ [13, 19, 20]	51 (0.7%)^a^
Gastric cancer	26.42 [13]	4.61 [13]	Advanced gastric cancer	1618 (5%)^a^ [13]	1177 (26.1%)^a^ [13]	477 (6.2%)^a^
Prostate cancer	207.87 [13]	16.82 [13]	Advanced prostate cancer	15,536 (48.4%)^a^ [13]	1257 (27.9%)^a^ [13]	3639 (47.2%)^a^
Multiple myeloma	10.09 [13]	1.79 [13]	Multiple myeloma	3929 (12.2%) [13]	677 (15%) [13]	1777 (23.1%) [21]
Total				32,119 (100%)	4510 (100%)	7703 (100%)

*Note:* Results show the absolute number of patients and the percentage of the total prevalent, incident, and eligible population. Authors calculations with data on prevalent and incident population from CAC [13]; incidence data is from IARC [17]. Data for the funnel‐down calculations are from the literature; superindex corresponds to the specific reference used.

Abbreviations: ALK, anaplastic lymphoma kinase; BC, breast cancer; CT, clinical trial; EGFR, epidermal growth factor receptor; HER2, human epidermal growth factor receptor 2; NSCLC, non‐small cell lung cancer.

^a^ CTIC's expert opinion was used when necessary.

### 3.2 Economic Impact

During the study period, experts independently identified the number of patients treated in the CTIC for prioritized tumors: 13 patients with advanced gastric cancer, 21 with non‐driver non‐small cell lung cancer (NSCLC), 11 with epidermal growth factor receptor (EFGR) NSCLC, and 32 with multiple myeloma. They then assessed how well the healthcare resources used by these patients within the CTIC reflected standard clinical protocol‐based management. Although some patients were treated for advanced HER2+ and triple‐negative breast cancer, advanced prostate cancer, and ALK and ROS1 NSCLC, experts deemed them unrepresentative in terms of healthcare resource utilization. Consequently, we estimated the average healthcare resource consumption per patient based on CTIC clinical protocols, ensuring alignment with the IETS BIA methodology.

Table 3 presents the estimated average cost per patient for each tumor type. Among them, advanced HER2+ breast cancer had the highest annual average treatment cost (USD 67,685), followed by ALK and ROS1 NSCLC at USD 65,316 and USD 46,176, respectively. The weighted average cost per patient was estimated at USD 31,695. If all eligible patients participated in CTs, the total cohort cost would be USD 244,151,552. Although advanced prostate cancer is not the most expensive condition in terms of per‐patient cost, it has the largest target population and the highest inclusion probability rates, accounting for approximately 48.6% of the total cost. Similarly, multiple myeloma represents 21.7% of the total cost.

**TABLE 3:** Estimated total cohort cost.

Tumor		Estimated drug cost per patient (USD)	Estimated cost per patient (non‐drug related)	Average patient cost (USD)	Eligible population for CT (*N*)	Total cohort cost
Breast cancer	Advanced HER2+ BC	62,261 (92%)	5424 (8%)	67,685	156	10,558,908 (4.3%)
	Advanced triple negative BC	9534 (38%)	15,301 (62%)	24,835	124	3,079,542 (1.3%)
Lung cancer	No driver NSCLC	23,544 (73%)	8550 (27%)	32,094	1035	33,217,628 (13.6%)
	EFGR NSCLC	23,865 (75%)	7968 (25%)	31,833	344	10,950,386 (4.5%)
	ALK NSCLC	54,729 (84%)	10,587 (16%)	65,316	100	6,531,553 (2.7%)
	ROS1 NSCLC	34,351 (74%)	11,826 (26%)	46,176	51	2,354,987 (1.0%)
Gastric cancer	Advanced gastric cancer	6705 (55%)	5540 (45%)	12,246	477	5,841,134 (2.4%)
Prostate cancer	Advanced prostate cancer	26,433 (81%)	6169 (19%)	32,602	3639	118,640,419 (48.6%)
Multiple myeloma	Multiple myeloma	19,641 (66%)	10,168 (34%)	29,809	1777	52,976,994 (21.7%)
All tumors		24,015	7680	31,695	7703	244,151,552 (100%)

*Note:* Drug and non‐drug related costs per patient are presented in absolute terms and proportional to the average cost per patient by tumor subtype. “All tumors” row represents the weighted average by eligible population. Total cohort cost in parentheses shows the share of the total cost for each tumor.

Abbreviations: ALK, anaplastic lymphoma kinase; BC, breast cancer; CT, clinical trial; EGFR, epidermal growth factor receptor; HER2, human epidermal growth factor receptor 2; NSCLC, non‐small cell lung cancer.

On average, drug costs constituted approximately 71% of the total expenditure (Table 2). Advanced triple‐negative breast cancer had the lowest proportion of drug‐related costs (38%), whereas advanced HER2+ breast cancer had the highest (92%).

### 3.3 Cohort Size Simulation

If enrollment rates reached 100% annually over 5 years, the Colombian healthcare system could achieve cost savings of USD 1.22 billion. However, given the challenges associated with patient enrollment in CTs, we estimated potential savings across different enrollment rates. Even with a target of enrollment of 20% of all potential candidates, annual savings would amount to approximately USD 48.8 million. In a scenario where enrollment rates increase by 20% each year, total cost savings could reach USD 244.2 million by the fifth year.

### 3.4 Deterministic Sensitivity Analysis

The sensitivity analysis demonstrated slight variability in the results, as presented in Table 4. Adjusting the eligible population for CTs based on the minimum and maximum prevalence of each disease led to a 9.5% reduction and a 1.6% increase, respectively. Consequently, the estimated savings ranged from USD 207 million to USD 235 million, reinforcing the robustness of the base case results. Regarding cost inputs, the analysis varied with the discount applied to drug costs. Even with a 30% reduction in pharmacological expenses, the inclusion of patients in CT resulted in savings of USD 190 million. Overall, both sensitivity analyses indicated that despite variations in key parameters, the inclusion of eligible patients in sponsored CT would lead to substantial cost savings for the Colombian healthcare system.

**TABLE 4:** Deterministic sensitivity analysis results, total cohort cost (USD) with variation in prevalence and costs.

Tumor subtype	Prevalence				Medication cost	
	CT elegible LV	CT elegible HV	Cost LV (USD)	Cost HV (USD)	Cost − 10% drug cost (USD)	Cost − 30% drug cost (USD)
Adv HER2+ BC	155	157	10,491,223	10,626,594	9,587,640	7,645,102
Adv triple negative BC	124	125	3,079,542	3,104,377	2,834,962	2,345,800
No driver NSCLC	1014	1056	32,543,647	33,891,609	30,780,832	25,907,241
EFGR NSCLC	337	352	10,727,559	11,205,047	10,129,436	8,487,535
ALK NSCLC	98	103	6,400,922	6,727,500	5,984,263	4,889,682
ROS1 NSCLC	50	52	2,308,811	2,401,163	2,171,573	1,804,746
Adv gastric Ca	473	481	5,792,152	5,890,116	5,521,287	4,881,593
Adv prostate Ca	3611	3667	117,727,549	119,553,288	109,681,251	91,762,915
Multiple myeloma	1721	2439	51,291,277	72,701,143	49,486,300	42,504,911
Total	7703	8432	240,362,683	266,100,837	226,177,543	190,229,526

*Note:* The 95% CI in the estimation of the prevalence rate was used for the prevalence low and high input values.

Abbreviations: Adv, advanced; ALK, anaplastic lymphoma kinase; BC, breast cancer; CT, clinical trial; EGFR, epidermal growth factor receptor; HER2, human epidermal growth factor receptor 2; HV, high input value; LV, low input value; NSCLC, non‐small cell lung cancer.

## 4 Discussion

Healthcare systems, particularly in low‐income and middle‐income countries, can achieve significant cost savings by enrolling patients in CTs, contributing to long‐term sustainability. In sponsored CTs, not only are drugs provided at no cost, but expenses related to diagnostic procedures, laboratory tests, and specialist follow‐up are also covered by the sponsor [22, 23]. To our knowledge, this is the first study in Colombia to use RWD to conduct an economic analysis of participation in privately sponsored CTs. Our findings estimate potential savings for the Colombian healthcare system at USD 244,151,552 per year. Globally, there is a growing interest in quantifying the economic benefits of CT participation.

Multiple studies have reported findings consistent with ours, demonstrating that CTs can lead to substantial cost reductions across various healthcare systems, ranging from publicly funded models, such as those in Canada and the United Kingdom (UK), to private insurance‐based systems like that of the United States [24, 25, 26]. In a retrospective cohort study using electronic medical records, Merkhofer et al. found that participation in second‐line CTs for metastatic NSCLC was associated with monthly savings of USD 6663 for US healthcare payers [27]. A study in the UK evaluating the treatment cost of 357 patients enrolled in research studies reported total savings of £885,275 over 2 years, averaging £9294 per patient [24]. Additionally, the inclusion of unsponsored academic clinical trials may generate cost savings in certain contexts or may not lead to substantial increases in treatment expenses [24]. Similarly, a Canadian study assessing the avoided costs of drug dispensing for patients enrolled in 101 clinical trials at an academic centre found potential cost savings of up to CAD 46,640 per patient [25].

Consistent with our findings, a retrospective study conducted at a Spanish tertiary hospital reported a significant cost reduction of € 696,002 in the healthcare system for patients with prostate cancer enrolled in CTs. Covering the period from 1996 to 2013, this study primarily included drugs, such as androgen deprivation therapy (ADT) and supportive medications, such as denosumab or bisphosphonates, which contributed to substantial economic benefit [28]. Similarly, our study observed higher estimated drug costs due to the inclusion of contemporary therapies, such as androgen receptor pathway inhibitors (ARPI) and PARP inhibitors, which are considerably more expensive than those examined by Calvin‐Lamas et al. [28].

Research consistently indicates that the sponsor's provision of drugs is the primary driver of cost savings in CTs. Koçkaya et al. reported direct drug cost savings of USD 311 million for the Turkish healthcare system between 2006 and 2010 [29]. Similarly, in Taiwan, Shen et al. estimated savings of USD 11.2 million from supplied drugs, with average savings of USD 58,000 per CT and USD 3900 per patient [30]. Based on our estimations, assuming similar average costs per patient, a 100% CT inclusion rate, and a 5‐year population growth projection using the BIA methodology, total savings could reach approximately USD 1.22 billion. It is important to emphasize that our analysis extends beyond drug cost avoidance alone but also includes the cost savings resulting from using other healthcare resources as part of routine clinical practice management, which is not usually assessed in similar studies.

Additionally, CTs generate positive externalities, including increased employability of highly specialized professionals, timely access to otherwise unavailable or experimental treatments, and enhanced experience and scientific contribution of healthcare professionals to society [1]. Moreover, a systematic review found that conducting CTs enhances research capacity, generates savings for the involved health systems, and leads to better health outcomes. This improvement is attributed to the controlled clinical environment, which ensures timely and comprehensive patient treatment in CTs [31]. Furthermore, a recent study in Austria reported that every Euro (€) invested in CTs by the pharmaceutical industry yielded € 1.95 across the broader economy [32]. Similarly, in the US, an investment exceeding USD 15 billion in CTs resulted in an economic impact of more than USD 42 billion [33]. These findings demonstrate the clear benefits for countries promoting clinical trials. Not only do they show cost savings associated with participation in clinical studies, but the literature also indicates improved patient outcomes [34].

Notably, as previously discussed, even in unsponsored academic CT settings, the costs associated with treatment or care do not increase significantly and may be reduced. This finding serves as a compelling rationale for health systems and policymakers to reconsider their unfounded concerns regarding the potential risk of increased costs resulting from patient participation in clinical trials [24, 35].

A potential limitation of our results is that the treatment costs for advanced prostate cancer, accounting for 49% of the total potential savings, were estimated using CTIC's clinical protocols due to the limited representativeness of healthcare resource utilization among the identified patients. Despite efforts to estimate the cost of treating patients with advanced prostate cancer, there is little available evidence for direct comparison [36]. Furthermore, comparing treatment costs across countries [37, 38] is challenging due to variations in disease management and healthcare resource costs. Additional evidence is required to assess the generalizability of our findings to this specific patient population. Additionally, treatment cost calculations in this study were based on routine clinical practice at a single institution.

## 5 Conclusion

Healthcare systems must improve the efficiency of CTs and increase patient enrollment to maximize benefits for both patients and healthcare infrastructure. Achieving this requires addressing key barriers to conducting CTs, including limited financial and human resources, challenges within ethical and regulatory frameworks, an underdeveloped research environment, operational inefficiencies, and competing priorities [39]. These obstacles may contribute to the low participation rate of approximately 8% in cancer CTs within low‐ and middle‐income [40].

Our findings offer valuable insights into the strengths of CTs as a strategy to alleviate financial pressure on the Colombian healthcare system, enhance long‐term financial sustainability, and expand patients' access to innovative health technologies that improve clinical outcomes. This, in turn, supports a key objective of the Colombian Ministry of Health's Ten‐Year Public Health Plan: reducing avoidable mortality and its associated loss of life years, as well as minimizing avoidable morbidity and disability and their impact on healthy life expectancy [41].

## Author Contributions

**Leonardo Rojas:** conceptualization (equal), investigation (equal), supervision (equal), writing – review and editing (equal). **Natalia Sánchez:** conceptualization (equal), investigation (equal), project administration (equal), supervision (equal), writing – review and editing (equal). **Jorge Ceballos:** conceptualization (equal), data curation (equal), resources (equal), writing – review and editing (equal). **Antonio Robles:** conceptualization (equal), data curation (equal), formal analysis (equal), methodology (equal), validation (equal), writing – review and editing (equal). **Carlos A. Badillo:** conceptualization (equal), data curation (equal), formal analysis (equal), methodology (equal), software (equal), validation (equal), writing – original draft (equal). **Virginia Abello:** conceptualization (equal), investigation (equal), supervision (equal), writing – review and editing (equal). **Carlos Bonilla:** conceptualization (equal), investigation (equal), supervision (equal), writing – review and editing (equal). **William A. Mantilla:** conceptualization (equal), investigation (equal), supervision (equal), writing – review and editing (equal). **Jairo Zuluaga:** conceptualization (equal), investigation (equal), supervision (equal), writing – review and editing (equal). **Gilberto Lopes:** conceptualization (equal), investigation (equal), supervision (equal), writing – review and editing (equal). **Oscar Arrieta:** conceptualization (equal), investigation (equal), supervision (equal), writing – review and editing (equal). **Andrés F. Cardona:** conceptualization (lead), formal analysis (lead), investigation (lead), methodology (lead), supervision (lead), validation (lead), writing – original draft (lead), writing – review and editing (lead).

## Ethics Statement

This study did not require ethical committee approval.

## Conflicts of Interest

The authors declare no conflicts of interest.

## Data Availability

Data is available upon reasonable request, except for internal cost‐related information.
